# Comparative Study on the In Vitro Fermentation Characteristics of Three Plant-Derived Polysaccharides with Different Structural Compositions

**DOI:** 10.3390/foods15010137

**Published:** 2026-01-02

**Authors:** Xingyue Gao, Xinming Zhao, Jie Huang, Huan Liu, Jielun Hu

**Affiliations:** Key Laboratory of Bioactive Polysaccharides of Jiangxi Province, China-Canada Joint Laboratory of Food Science and Technology (Nanchang), State Key Laboratory of Food Science and Resources, Nanchang University, Nanchang 330047, China; 417900230190@email.ncu.edu.cn (X.G.); zxm18482104090@163.com (X.Z.); huang_jie2025@163.com (J.H.)

**Keywords:** plant polysaccharides, molecular weight, antioxidant activity, short-chain fatty acids, prebiotic

## Abstract

This study aimed to elucidate the structure–activity relationship between the structural characteristics of three plant-derived polysaccharides, Lycium barbarum polysaccharide (LBP), citrus pectin (CP) and peach gum polysaccharide (PGP), and their prebiotic functionalities. Structural analysis indicated that LBP exhibited a medium molecular weight and was rich in galactose and rhamnose, which contributed to its high uronic acid content, strong antioxidant activity, and sustained fermentation profile with enhanced butyrate production. In contrast, CP, with its low molecular weight and neutral linear glucan backbone, was rapidly utilized by gut microbiota, leading to accelerated propionate accumulation. Meanwhile, PGP, characterized by an ultra-high molecular weight and a highly branched arabinogalactan configuration, acted as a specific substrate that promoted mid- to late-stage fermentation and significantly increased butyrate yield, highlighting its prebiotic property driven by structural complexity. The functional differences among these polysaccharides were determined by their monosaccharide composition, molecular weight distribution, and chain conformation. These findings provide a scientific basis for the targeted development of plant-derived prebiotics aimed at specific metabolic functions.

## 1. Introduction

According to the International Union of Pure and Applied Chemistry (IUPAC), plant-derived polysaccharides are defined as naturally occurring high-molecular-weight carbohydrates extracted or isolated from plant tissues (including roots, stems, leaves, flowers, fruits, and seeds). They consist of polymers formed by more than ten monosaccharide or monosaccharide derivative units linked via glycosidic bonds. Extensive studies have demonstrated that polysaccharides exhibit a broad spectrum of bioactive properties, including antioxidant, antimicrobial, antitumor, hypoglycemic, anti-inflammatory, and immunomodulatory activities [1,2,3,4,5]. Predominantly composed of β-glycosidic bonds, plant-derived polysaccharides typically exhibit irregular and complex branched architectures along with diverse functional group modifications. These polysaccharides exhibit a wide range of molecular weights and degrees of polymerization, which may influence their selectivity toward specific bacterial communities. Compared to polysaccharides derived from animal sources (e.g., chitosan, hyaluronic acid) or those produced via microbial fermentation (e.g., xanthan gum), the complex structural features of plant-derived polysaccharides confer resistance to digestion, thereby supporting their potential role as prebiotics to stimulate microbial growth and promote the production of short-chain fatty acids (SCFAs). Meanwhile, plant-derived polysaccharides generally offer a range of advantages, including broader availability, relatively low cost, greater renewability, and higher consumer acceptance. Chen et al. [6] systematically evaluated the prebiotic effects of oat β-glucan using an in vitro fermentation model with human fecal microbiota and revealed that, compared to the control group, oat β-glucan significantly increased the relative abundance of *Bifidobacterium* and *Lactobacillus* and markedly enhanced the production of SCFAs, particularly propionate and acetate.

Prebiotic is defined as a substrate that is selectively utilized by host microorganisms, conferring health benefits on the host, such as enhancing mineral absorption, reducing the incidence of certain cancers, modulating metabolic processes, and promoting the production of SCFAs by the gut microbiota [7]. Currently developed prebiotics primarily comprise indigestible low-molecular-weight carbohydrates, such as fructooligosaccharides (FOS), inulin, resistant dextrin, and resistant starch. Among these usual prebiotics, FOS and inulin have been demonstrated to significantly promote the proliferation of beneficial gut bacteria including *Bifidobacterium* and *Lactobacillus*, while suppressing the growth of pathogenic bacteria [8]. In addition, they can be selectively utilized by gut microbiota to produce SCFAs, consequently reducing intestinal pH and optimizing the gut microecology [9]. Resistant dextrin and resistant starch mainly featured beneficial characteristics of regulating glycemic homeostasis and enhancing intestinal barrier function. Hobden et al. [10] systematically evaluated the physiological effects of resistant dextrin through a randomized double-blind controlled clinical trial and found that daily supplementation with resistant dextrin led to a moderated postprandial glycemic response, alongside a significant increase in bifidobacterial populations within the gut. Miketinas et al. [11] researched the physiological effects of resistant starch and found that daily supplementation with resistant starch significantly improved insulin sensitivity and increased the abundance of *Ruminococcus* bacteria in the human gut. However, the inherent structural complexity and diversity of plant-derived polysaccharides, combined with the influence of environmental factors on their prebiotic efficacy, make standardization a significant challenge. Consequently, the availability of available polysaccharide-derived prebiotics derived from these polysaccharides remains limited and, therefore, it has become crucial to systematically explore novel polysaccharides with prebiotic effects from natural resources to diversify the options for human daily intake.

This study first aimed to comprehensively characterize the physicochemical properties of three plant-derived polysaccharides with significant structural differences, lyceum barbarum polysaccharide (LBP), citrus pectin (CP) and peach gum polysaccharide (PGP), and to elucidate the structure–activity relationship between their distinctive molecular compositions and differentiated prebiotic activities. First, the fundamental physicochemical properties and molecular composition of these three plant-derived polysaccharides were characterized. Second, the fermentation characteristics of three polysaccharides were evaluated using an in vitro fermentation model. Finally, the prebiotic potential was further elucidated through quantitative profiling of SCFA production.

## 2. Materials and Methods

### 2.1. Materials

Fresh *Lycium barbarum* fruits were purchased from Ningxia Lycium barbarum Co., Ltd. (Zhongwei, China). LBP was extracted using hot water extraction. CP and PGP were sourced from Shanghai Yuanye Bio-Technology Co., Ltd. (Shanghai, China) and Jiangsu Ruiduo Biotechnology Co., Ltd. (Changzhou, China), respectively. Papain (EC 3.4.22.2), thermostable α-amylase (EC 3.2.1.1), and phenol were obtained from Shanghai Aladdin Biochemical Technology Co., Ltd. (Shanghai, China). Chromatographically pure D-galacturonic acid (≥99%) was supplied by Beijing Solarbio Science & Technology Co., Ltd. (Beijing, China). All other chemical reagents were analytical grade and purchased from Xilong Scientific Co., Ltd. (Shantou, China).

### 2.2. Component Analysis of Polysaccharides

The basic compositional profiles of LBP, CP, and PGP were characterized using established colorimetric assays. The basic compositional profiles of LBP, CP, and PGP were characterized using established colorimetric assays. The total neutral sugar content was estimated using the phenol–sulfuric acid method. A standard D-glucose solution (0.1 mg/mL) was used to prepare a series of diluted standards (0, 0.1, 0.2, 0.3, 0.6, 0.8, and 1.0 mL of the standard solution, each made up to 1 mL with distilled water). Then, 1 mL of 5% phenol and 5 mL of concentrated sulfuric acid were added to each tube. After mixing and incubation, the absorbance was measured at 490 nm to construct the standard curve. For sample analysis, 1 mL of a 0.1 mg/mL polysaccharide solution was processed following the same procedure. The acidic sugar (uronic acid) content was quantified via the carbazole–sulfuric acid method, calibrated with D-galacturonic acid using concentrations and dilutions consistent with the glucose standard. To each standard or sample, 6.0 mL of concentrated sulfuric acid was added, followed by incubation at 85 °C for 20 min in a water bath. Then, 0.2 mL of carbazole–ethanol solution was added, and, after mixing and standing, the absorbance at 530 nm was measured with a microplate reader to plot the standard curve. Sample measurements were performed identically. Meanwhile, the protein content was determined using the Bradford method, with bovine serum albumin (BSA) as the standard. Diluted BSA standards and samples were mixed with 5 mL of Coomassie Brilliant Blue reagent, and the absorbance was measured at 595 nm. The protein content of the samples was calculated based on the BSA standard curve [12,13].

### 2.3. Structural Characteristics Analysis of Polysaccharides

Fourier-Transform Infrared (FT-IR) spectra of LBP, CP, and PGP were acquired using Fourier infrared spectrometer (Nicolet 5700, Thermo Fisher Scientific, Waltham, MA, USA). Approximately 1 mg of the sample was thoroughly mixed with 100 mg of dried potassium bromide (KBr) and ground to micron-scale uniformity in an agate mortar. The mixtures were compressed into transparent pellets under 10-ton hydraulic pressure for 1 min. Spectra were recorded in transmittance mode across 4000-400 cm^−1^ at 4 cm^−1^ resolution with 32 scans per sample. Data processing was performed using OMNIC™ software (version 9.2.16).

### 2.4. Monosaccharide Composition and Molecular Weight Distribution of Polysaccharides Analysis

The monosaccharide constituents of the polysaccharides were analyzed via high-performance anion-exchange chromatography coupled with pulsed amperometric detection (HPAEC-PAD). Monosaccharide standards, including D-galacturonic acid (GalA), L-rhamnose (Rha), D-galactose (Gal), D-glucose (Glc), D-xylose (Xyl), D-arabinose (Ara), and D-mannose (Man), were purchased from Sigma-Aldrich. All standards were of chromatographic grade (≥98% purity). Briefly, samples (5 mg) were first subjected to two-step acid hydrolysis. They were initially solubilized in 0.5 mL of 12 M H_2_SO_4_ on ice, followed by dilution to a 2 M concentration and complete hydrolysis in a 120 °C oil bath for 2 h [14]. The hydrolysis reaction was quenched by immediate cooling in an ice-water bath. A 250-fold dilution of the hydrolysate with ultrapure water was performed, followed by filtration through a 0.22 μm membrane prior to HPAEC-PAD analysis. The analysis was carried out on a Dionex ICS-5000 system (Thermo Fisher Scientific, Waltham, MA, USA) equipped with a CarboPac™ (version 7.3) PA20 guard column (3 × 30 mm) and an analytical column (3 × 150 mm) with operational parameters set as follows: flow rate of 0.5 mL/min, column temperature of 30 °C, detector temperature of 35 °C, and injection volume of 10 μL.

Molecular weight distribution was analyzed using gel permeation chromatography (GPC) following a previously described method with minor modifications [15]. An Agilent 1260 High-Performance Liquid Chromatography System equipped with a Waters Ultrahydrogel™ 250 linear column (7.8 × 300 mm, Waters, Milford, MA, USA) was used. The analyses were conducted at 30 °C with a 0.02% NaN_3_ solution as the mobile phase at a flow rate of 0.6 mL/min, and samples prepared at a concentration of 1.0 mg/mL in the same solvent were injected at a volume of 20 μL. A series of pullulan standards were used for molecular weight calibration [16].

### 2.5. Morphological Characteristics Analysis of Polysaccharides

The morphological characteristics of LBP, CP, and PGP samples were investigated using a scanning electron microscope (SEM, SU8010, Hitachi Ltd., Tokyo, Japan). Specifically, an appropriate amount of sample powder was placed on the conductive adhesive, and the excess polysaccharide sample was gently removed using a rubber bulb. In order to improve the conductivity of the sample, these powders were subjected to gold sputtering (Hitachi E-1010, Tokyo, Japan) before being transferred to SEM for morphological characteristic analysis. Images were taken at both low (500×) and high (5000×) magnifications using an accelerating voltage of 5 kV.

### 2.6. Antioxidant Activity Analysis

The antioxidant activity of LBP, CP and PGP samples was evaluated by using 2,2-diphenyl-1-picrylhydrazyl (DPPH) radical and 2,2′-azino-bis(3-ethylbenzothiazoline-6-sulfonic acid) (ABTS^+^•) radical cation removal experiments [17], with vitamin C (Vc) serving as a positive control, and corresponding improvements were made according to the methods described. For the evaluation of free radical removal ability, a polysaccharide solution with a concentration in the range of 0.5 to 4 mg/mL was prepared. Aliquots (1 mL) of polysaccharide solutions were mixed with 0.6 mM DPPH or 7 mM ABTS solutions in test tubes and incubated in darkness for 30 min (DPPH) or 10 min (ABTS). The positive control group (Vc) was processed under identical conditions to validate the experimental procedure and provide a benchmark for activity comparison. The antioxidant potential of the samples was evaluated by their ability to scavenge DPPH and ABTS radicals. For the DPPH assay, the decrease in absorbance was monitored at 517 nm. Similarly, the ABTS scavenging activity was assessed by measuring the absorbance decay at 734 nm. Both measurements were performed using an Agilent microplate reader [18].

The percentage of radical scavenging was estimated based on the reduction in absorbance of the radical solution, calculated with Equation (1).

(1) $$\mathrm{Scavenging}\;\mathrm{activity}\;\left(\%\right)=\frac{A_{1}-\left(A_{S}-A_{0}\right)}{A_{1}}\times 100\%$$

where A_s_: absorbance value of the solution after sample reaction; A_0_: absorbance value of the solution without reaction; A_1_: absorbance value of the solution without sample addition.

### 2.7. In Vitro Fermentation of Polysaccharides

#### 2.7.1. Colony Collection and In Vitro Fermentation Modelling

An anaerobic basal medium was prepared as described previously [19], containing (g/L): yeast extract (2), peptone (2), NaHCO_3_ (2), bile salts (0.5), Tween-80 (2), L-cysteine hydrochloride (0.5), resazurin (0.5), and a mineral salt solution. Fresh fecal samples for in vitro fermentation were collected from six healthy volunteers (three females and three males) with no history of diarrhea or antibiotic use in the preceding three months. Upon collection, fresh fecal samples should be transferred within 2 h to an anaerobic chamber (COY Laboratory Products, USA) and homogenized in sterile 0.1% (*w*/*v*) L-cysteine-PBS buffer at a 1:5 (*w*/*v*) ratio to create a fecal slurry. The fecal suspensions were vigorously vortexed and filtered through sterile cell strainers to obtain individual fecal filtrates. Equal volumes of individual filtrates were subsequently pooled to generate a mixed fecal filtrate.

Prior to fermentation, 0.12 mL of a vitamin K_1_-hemin mixture was aseptically transferred into anaerobic tubes and homogenized. Subsequently, 0.12 mL of fecal filtrate was added, vortex-mixed, and subjected to batch fermentation under anaerobic conditions for 48 h. Samples were collected at five time points (0, 6, 12, 24, and 48 h) for subsequent determination of relevant experimental parameters.

#### 2.7.2. Measurements of Gas Production, OD_600_, pH, and Sugar Content During the Fermentation Process

Triplicate samples were aspirated at predetermined time points using a syringe, with aspiration volumes determined by the final resting position of the syringe plunger, and the final gas volume was determined. Aliquots of fermentation broth were analyzed for optical density at 600 nm (OD_600_) using a microplate reader and for pH using a calibrated pH meter. A Mettler Toledo LE438 combination pH electrode (Cat. No. 51340242) was employed for all measurements. Additionally, 50-fold ultrapure water-diluted fermentation broth samples were subjected to sugar content quantification via the phenol–sulfuric acid method against glucose standards.

#### 2.7.3. Determination of SCFAs During Fermentation

The content of SCFAs, comprising acetic acid, propionic acid, and butyric acid, among others, was determined by Gas Chromatography–Flame Ionization Detector (GC-FID) analysis [20]. A calibration curve was established using SCFA standards (Supelco CRM46975, Bellefonte, PA, USA) to enable quantitative analysis. The sample preparation and chromatographic conditions were as follows: Sample Workup: fecal fermentation broth was centrifuged (13,000 rpm, 5 min, 4 °C), and the supernatant was double-filtered through 0.22 μm membranes. Aliquots of filtrate (0.65 ± 0.05 mL) were mixed with 0.2 mL of 10% (*v*/*v*) concentrated sulfuric acid, vortexed for 1 min, then spiked with 10 μL internal standard and 0.4 mL anhydrous ethyl ether. GC-FID Analysis: metabolite separation and quantification were carried out on an Agilent 7890 GC system equipped with an HP-5 capillary column and an FID. The method utilized a 1 μL injection at 200 °C with a 10:1 split ratio. Nitrogen served as the carrier gas (1 mL/min). The oven temperature was initiated at 50 °C and increased to 230 °C at a ramp of 10 °C/min [21].

### 2.8. Data Analysis

Data are expressed as mean ± standard deviation (SD). Prior to assessing group differences, the data were subjected to one-way analysis of variance (ANOVA). When a significant effect was found, Tukey’s post hoc test was applied for multiple comparisons, with the significance threshold set at *p* < 0.05 [22].

## 3. Results and Discussion

### 3.1. Composition Analysis of Different Polysaccharides

**Figure 1:**
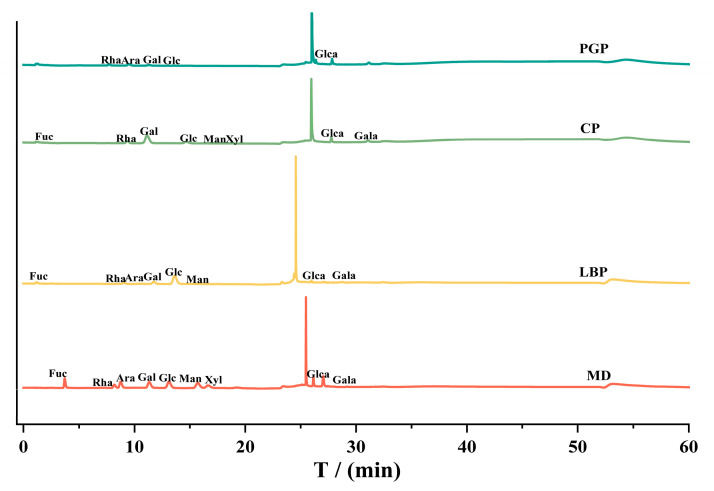
HPAEC-PAD chromatogram of different polysaccharides. MD: Monosaccharide standards.

The monosaccharide compositions of different polysaccharides are shown in Figure 1, and the corresponding data are summarized in Table 1. All three polysaccharides were identified as heteropolysaccharides composed of rhamnose (Rha), galactose (Gal), glucose (Glc), etc. Specifically, LBP was predominantly composed of Gal (33.08%) and Rha (21.73%). The high levels of galactose and rhamnose in LBP were primarily due to its rich content of rhamnogalacturonan I (RG-I) pectin, with galactose concentrated within the branched side chains. The high molar ratios of these two monosaccharides collectively underpin the significantly elevated acidic sugar content (36.76 ± 4.63%) observed in LBP, which was more than three-times higher than that of both CP and PGP (Table 2). CP was predominantly composed of Glc (70.77%) and Man (14.02%), with only trace amounts of Gala (1.24%). The high proportion of Glc indicated that the structural framework of CP was predominantly composed of glucan or cellulose-like backbones. Concurrently, the presence of Man suggested the inclusion of neutral polysaccharide components, such as mannan or galactomannan. These monosaccharide profiles correlated well with the notably high neutral sugar content (87.05 ± 0.42%) and low acidic sugar content (9.34 ± 0.64%) (Table 2). In contrast, PGP was predominantly composed of Gal (45.18%) and Ara (32.56%), with a minor portion of Glca (2.15%). The high abundance of galactose (Gal) and arabinose (Ara) indicated that the structural framework of PGP was a highly branched arabinogalactan. Concurrently, the minimal presence of glucuronic acid (Glca) suggested that acidic residues contribute negligibly to the overall structure, which was consistent with the measured high neutral sugar content (80.06 ± 0.15%) and low acidic sugar content (6.61 ± 0.12%) (Table 2).

### 3.2. Structural Characteristics Analysis of Different Polysaccharides

**Figure 2:**
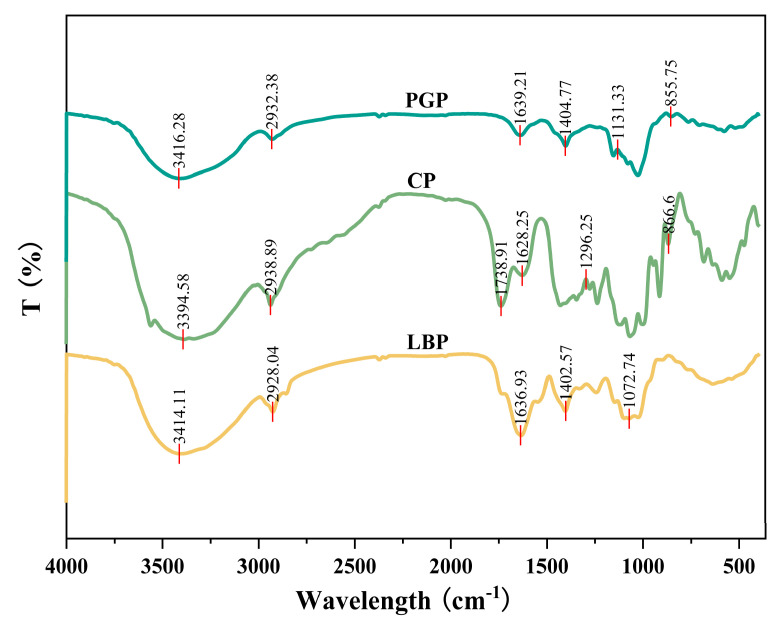
FT-IR spectra of different polysaccharides (characteristic peaks at specific wavenumbers are indicated by the red lines).

The FT-IR spectra of different polysaccharides are shown in Figure 2. The FT-IR spectra of LBP, CP, and PGP exhibited characteristic polysaccharide absorption bands in the range of 4000–500 cm^−1^ along with distinct vibrational modes. Common infrared features among these three polysaccharides included a broad and intense peak around 3400 cm^−1^, attributed to O–H stretching vibrations arising from abundant hydroxyl groups and intermolecular hydrogen bonds [23]; relatively weak absorption near 2900 cm^−1^, corresponding to C–H stretching vibrations primarily from methylene and methine groups; absorptions around 1640 cm^−1^ and 1400 cm^−1^, associated with O–H bending vibrations of bound water and C–H deformation vibrations, respectively; a strong and broad absorption band in the 1000–1100 cm^−1^ region, resulting from overlapping signals of C–O–C and C–O stretching vibrations of pyranose rings, which served as a typical marker of the polysaccharide backbone structure [24]. Specifically, for LBP, a characteristic absorption band at approximately 1072 cm^−1^ was observed, attributable to the C–H out-of-plane bending vibration of β-glycosidic linkages. This observation was consistent with its high content of glucose and galactose, which typically adopt β-configurations, suggesting a backbone structure predominantly composed of β-glycosidic bonds. In addition, the distinct absorption bands observed near 860 cm^−1^ for both CP and PGP can be assigned to the characteristic out-of-plane bending vibrations of C-H bonds on pyranose rings, typically associated with monosaccharides such as mannose or galactose [25].

### 3.3. Morphological Characteristics Analysis of Different Polysaccharides

**Figure 3:**
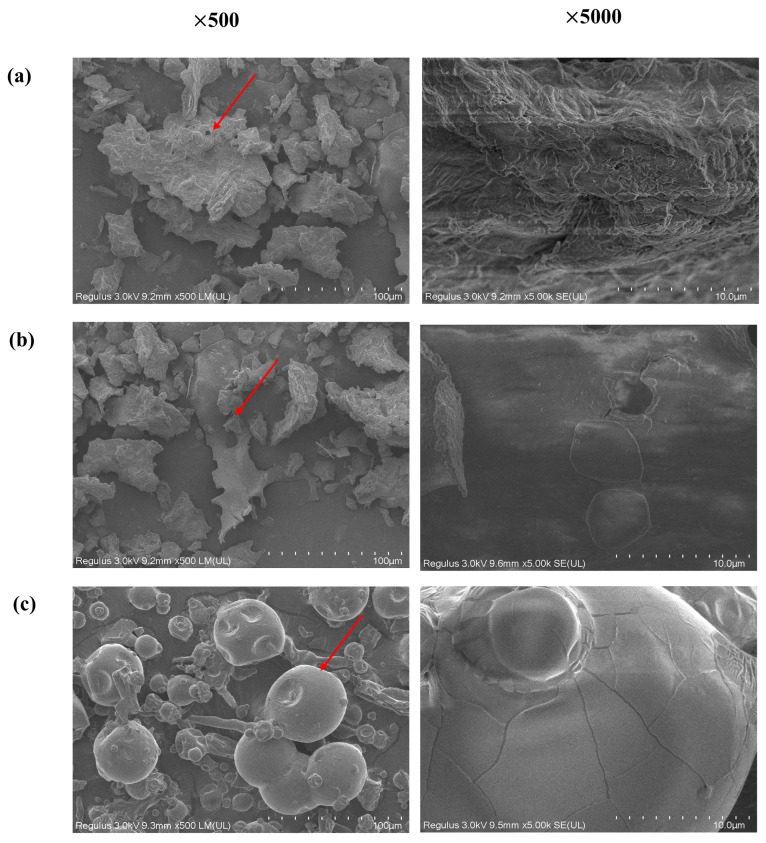
SEM images of different polysaccharides: (**a**) LBP; (**b**) CP; (**c**) PGP. The arrows in the figure indicate the three distinct morphological features of the polysaccharides.

The apparent morphological features of different polysaccharides are observed using SEM (Figure 3). LBP and CP showed obviously flaky surface morphology and high surface density, which could be mainly attributed to a large amount of inter- and intramolecular hydrogen bond formation during extraction and drying. It was the typical feature of plant cell wall polysaccharides. The linear or slightly branched chain morphology of these polysaccharides facilitated the close molecular packing and two-dimensional extension, which led to the layered structure [26]. In contrast to LBP, CP exhibited a smoother and more continuous lamellar surface morphology, primarily attributable to its simpler monosaccharide composition and the resultant linear molecular conformation. Because the monosaccharide composition of CP mainly consisted of Glc (70.77%) and Man (14.02%) with an exceptionally low acidic sugar content (9.34%) (Table 1), the above structural characteristics indicated that CP might have a molecular structure with a glucan- or cellulose-like linear characteristic, which might induce highly regular and oriented molecular assembly during the drying process [27], which would further promote the formation of compact and ordered lamellar stacking with extensive intermolecular hydrogen bonding, finally leading to the continuous and smooth surface morphology [28]. Based on the analysis of weight-average molecular weight (Mw) and Table 2, the macro-morphology of PGP was irregularly developed with many depressions on the sphere surface, which could be mainly caused by the extremely high Mw of PGP (37.92 × 10^4^ g·mol^−1^) in this experiment. The high Mw of PGP suggested the existence of a high degree of chain entanglement and strong interactions between PGP chains. When solvent evaporation and film formation process took place, the high Mw fractions of PGP experienced an uneven contraction process and orderly packing of high Mw fractions was hindered, finally leading to macro-morphological heterogeneity of PGP. The depressions on the sphere surface were usually formed by stress concentration field and interfacial phase separation behavior induced by the fast evaporation of the solvent in the vacuum or freeze-drying process. In addition, PGP was a kind of polyanionic polysaccharide, so electrostatic repulsion between the PGP chain also hinders close packing of PGP chain segments, which further leads to the heterogeneity of PGP surface morphology [29].

### 3.4. Antioxidant Activity Analysis of Different Polysaccharides

**Figure 4:**
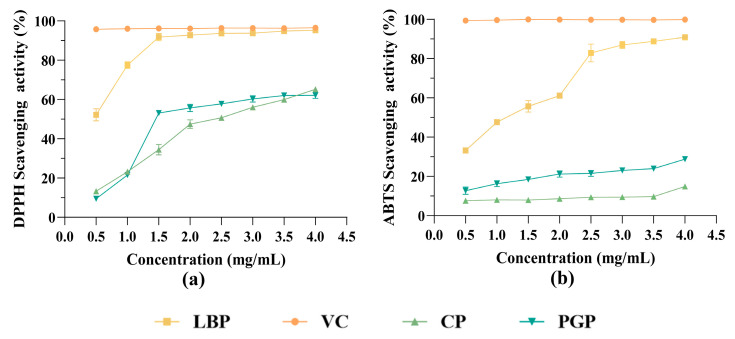
Antioxidant activities of different polysaccharides: (**a**) DPPH radical scavenging activity; (**b**) ABTS radical scavenging activity.

The DPPH and ABTS radical scavenging activities of different polysaccharides are shown in Figure 4. The DPPH radical scavenging assay is shown in Figure 4a. All three kinds of polysaccharides presented concentration-dependent antioxidant activities, and scavenging rates increased significantly with the increase in polysaccharide concentration. This may be mainly due to the larger number of active hydroxyl and phenolic hydroxyl groups as hydrogen donors to effectively reduce stable DPPH radicals available at higher polysaccharide concentrations [30]. LBP demonstrated significantly higher scavenging rates than CP and PGP across all concentration points, approaching the efficiency of Vc at concentrations above 2.0 mg/mL. This superior performance could be attributed to its moderate Mw and, more significantly, to its unique monosaccharide composition—characterized by high levels of galactose and rhamnose, coupled with a substantial acidic sugar content (36.76%) (Table 1). These acidic sugar units (e.g., galacturonic acid) introduced negatively charged functional groups such as carboxyl groups, which enhanced electron-donating capacity and consequently improved the efficiency of radical reduction [31].

For the ABTS^+^• radical scavenging assay (Figure 4b), CP and PGP consistently showed lower scavenging activities and responded poorly to concentration increases. LBP, however, displayed dramatically higher and concentration-dependent scavenging rates under the same conditions. This discrepancy primarily stemmed from the fundamental differences between these two radical systems and the structural compatibility of the polysaccharides [32]. The ABTS^+^• cation is a water-soluble radical whose quenching mechanism relies more heavily on the electron-donating capacity and efficient molecular diffusion of antioxidants in the aqueous phase. Conversely, the DPPH radical is hydrophobic, with its reaction favoring a hydrogen atom transfer mechanism typically occurring in organic media. CP, predominantly composed of neutral, linear glucan structures, lacked strong electron-donating groups (e.g., acidic sugars or phenolic hydroxyls), thereby abolishing its ability to reduce ABTS^+^• [33]. Although PGP possessed a similar anionic character, due to its low uronic acid content, the extremely high molecular weight and highly branched architecture severely restricted molecular mobility and accessibility of active sites in the aqueous phase, leading to inefficient electron transfer [34]. In contrast, the high acidic sugar content (36.76%) in LBP conferred a distinct polyanionic character, thereby promoting electrostatic attraction with ABTS^+^• and facilitating electron transfer in the aqueous phase [35].

### 3.5. In Vitro Fermentation Analysis of Different Polysaccharides

**Figure 5:**
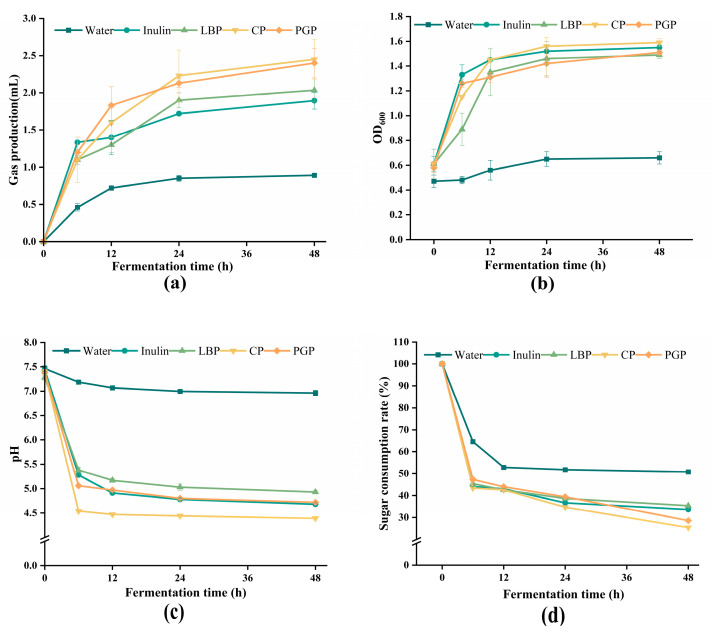
Changes in (**a**) gas production; (**b**) OD_600_ values; (**c**) pH; (**d**) sugar content of different polysaccharides during in vitro fermentation.

Carbohydrates are readily fermented by gut microbiota, generating various gases, including H_2_, CH_4_, CO_2_, and NH_3_ [36], and the volume of gas produced during fermentation serves as a key indicator of the metabolic activity of gut microorganisms toward the substrate. As shown in Figure 5a, the fecal microbiota cultured with different substrates exhibited differential gas production profiles throughout the fermentation process. The gas generation observed in the control group (water) reflected the baseline metabolic activity caused by residual endogenous substrates. During the initial 0–6 h of fermentation, all polysaccharide groups and the inulin control demonstrated rapid gas production compared to the water. It is worth noting that the gas production rate of inulin is significantly higher than that of other substances, which is caused by its simple structure. This simple structure enabled rapid enzymatic recognition and degradation by a broad range of gut bacteria, leading to the swift generation of H_2_ and CO_2_ [37]. In the next 6 to 48 h of fermentation, the three groups of polysaccharides significantly increased the gas yield, and at the 12th hour, their yield exceeded that of the inulin group. At 48 h, the cumulative gas volumes for LBP, CP, and PGP reached 2.03 ± 0.15, 2.45 ± 0.27, and 2.40 ± 0.20 mL, respectively, which was significantly higher than that in the inulin group (1.89 ± 0.11 mL). This continuous gas production phenomenon highlights the characteristics of the long-term fermentation process of polysaccharides, which stems from its complex and diverse glycosidic bond structures, such as β-pyran glycosidic bonds, α configurations and potential branch patterns, which require a long microbial adaptation period and the induction time of specific enzymes [38]. Once decomposed by a specific microbiome, the rich monosaccharide composition and chain structure inside the polysaccharide can be continuously utilized, thus supporting a longer gas generation process [39].

OD_600_ serves as a real-time surrogate indicator of microbial growth, quantifying the turbidity of the culture medium to indirectly reflect cumulative biomass accumulation. Consequently, its temporal profiles exhibit direct correlations with the prebiotic activity of polysaccharides [40]. As shown in Figure 5b, compared with the water group, the OD_600_ value of the polysaccharide group and the inulin group rose rapidly between 0 and 12 h, indicating that there was a stage of the rapid proliferation of microorganisms. Between 12 and 48 h, the OD_600_ value continued to rise slowly without significant differences and finally reached a similar stable level at 48 h, indicating that these polysaccharides and inulin have a similar role in promoting microbial growth and can achieve considerable biomass accumulation [41].

The dynamic decline in pH serves as a direct biomarker of microbial metabolic activity, which exhibits strong associations with both the prebiotic efficacy of polysaccharides and consequential gut health outcomes. As shown in Figure 5c, during the fermentation period of 0 to 6 h, the pH values of the polysaccharide group and the inulin group decreased rapidly and then tended to stabilize. At the end stage of fermentation, the pH value of the CP group was the lowest (4.39 ± 0.03), which can be attributed to its low molecular weight and relatively simple linear structure, which is conducive to the degradation of microorganisms and can produce more acidic metabolites [42]. The final pH value of the PGP group was comparable to that of the inulin group; however, the final pH value of the LBP group was significantly higher (4.93 ± 0.04). This phenomenon can be attributed to the low content of easily fermentable neutral sugars in LBP, especially the decrease in the content of Arabic sugar and glucose, which directly slows down microbial fermentation, which leads to an increase in pH.

In the process of in vitro fermentation, the dynamic decline in the carbohydrate content can be a clear sign of the utilization efficiency of microbial substrates. Its consumption trajectory directly reflects the structural accessibility of polysaccharides, the metabolic preferences of intestinal microorganisms and the functional characteristics of prebiotics [43]. As shown in Figure 5d, all samples showed rapid carbohydrate consumption within the first 0 to 6 h, while the water sample had almost no carbon source consumption, which may be due to its complete dependence on the residual carbon source in the culture medium. Between 6 and 24 h, the three polysaccharides and inulin demonstrated parallel consumption kinetics, reflecting efficient microbial utilization of the substrates under nutrient-sufficient conditions. In the 24–48 h stage, the residual sugar rates of CP and PGP were 25.4% and 28.6%, respectively, and their consumption rate was 10–15% faster than that of inulin, while the LBP group was about 8% slower. These various consumption dynamics primarily stemmed from the molecular structural characteristics of different polysaccharides. The low-molecular-weight fragment in CP is quickly utilized within 0–24 h, and the residual structure is a highly linearized, short side-chain ratose galactose aldehyde glycoglycan-I (RG-I) main chain structure. The structure is always in the action area of β-galactosidase, thus effectively promoting the enzymatic process of probiotics. PGP is a structure composed of a high-molecular-weight α-1,6-glucan bone, and its Arabic sugar side chain is consumed first, which makes it easier for the glucanase from *Mycobacterium* to contact the core glucan chain, thus promoting the rapid glycolysis process.

### 3.6. SCFAs Production Analysis During Fermentation

**Figure 6:**
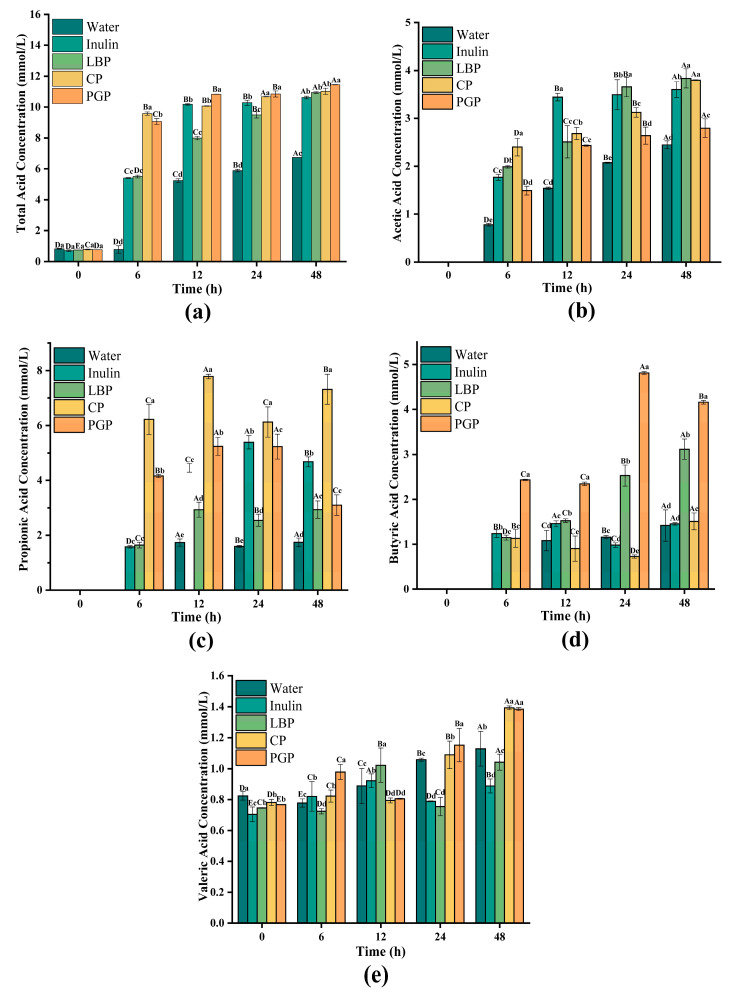
SCFAs production during in vitro fermentation of different polysaccharides: (**a**) total acid; (**b**) acetic acid; (**c**) propionic acid; (**d**) butyric acid; (**e**) valeric acid. Different letters indicated statistical significance at *p* < 0.05. Capital letters indicated significant differences between groups for the same polysaccharide at different fermentation times, while lowercase letters indicated significant differences within groups for different polysaccharides at the same fermentation time.

The anaerobic fermentation of carbohydrates by the gut microbiota yields SCFAs. These compounds exert a profound influence on the host’s physiology, contributing to the regulation of gut microbial ecology, the preservation of epithelial barrier function, and the modulation of systemic immune responses [44]. The principal SCFAs comprise acetate, propionate, butyrate and valeric acid [45]. Acetate serves as a key energy substrate for peripheral tissues, and elevated acetate concentrations (>50 mM) can inhibit colonization by pathogens such as *Salmonella* [46]. For valerate production (Figure 6e), the water group’s concentration rose step by step from 0.82 to 1.13 mmol/L. This was higher than the inulin group (consistently under 1 mmol/L) and also higher than the LBP group [47]. Butyrate, the primary energy substrate for colonic epithelial cells, reinforces epithelial barrier integrity through the activation of histone deacetylase (HDAC) inhibitors. Valproate exerts an immunomodulatory role and maintains the integrity of the intestinal barrier through the GPR41/GPR43 signaling pathway. At higher concentrations, it can also promote the secretion of glucagon-like peptide-1, thus improving glucose homeosis. The dynamic changes in SCFAs in the 48 h *in vitro* simulation fermentation of different polysaccharides are shown in Figure 6. Total acid contents of all samples accumulated progressively over time (Figure 6a). The water group maintained a consistently low level of total acid production, increasing gradually from an initial 0.82 mmol/L to a final concentration of 6.73 mmol/L, reflecting limited carbon source availability and low microbial metabolic activity in the basal medium. The inulin group exhibited rapid acid accumulation, reaching 10.17 mmol/L by 12 h, after which the rate of increase decelerated, culminating in a final concentration of 10.62 mmol/L. This pattern aligned with the metabolic characteristics of a readily fermentable substrate, potentially associated with the rapid proliferation of probiotics such as *Bifidobacterium*. Among the three polysaccharide groups, LBP showed a steady and sustained increase in total acid, rising from an initial 0.75 mmol/L to a final level of 10.93 mmol/L. Although CP and PGP reached similar final total acid yields (11.02 mmol/L and 11.44 mmol/L, respectively), CP demonstrated a faster initial increase, achieving 9.57 mmol/L by 12 h, which can be attributed to its linear backbone and easily cleavable glycosidic bonds. In contrast, PGP exhibited a more gradual fermentation profile, which can be due to its branched architecture and high molecular weight that initially restricted the accessibility and efficiency of relevant enzyme systems.

Specifically, in the case of acetate (Figure 6b), the inulin group increased rapidly within 0–12 h and then entered the platform period, and the accumulation rate slowed down, which may be due to the early depletion of its highly fermentable and degradable components. In contrast, the three polysaccharide groups demonstrated a slow yet sustained rise in acetate production throughout the fermentation period. At 4 h, the acetate level of the LBP group and the CP group was comparable to that of the inulin group, while the PGP level was significantly lower than that of the other two groups, which may be due to their structural complex characteristics, such as the high branching structure or different connection types, which will delay the microbial degradation process. Regarding propionate (Figure 6c), the inulin and PGP groups exhibited an initial increase followed by a decline, potentially reflecting metabolic shifts or substrate preference changes in propionate-producing bacteria such as *Propionibacterium* and *Veillonella*. The temporal profile of propionate revealed distinct fermentation patterns among the substrates. While LBP supported a steady increase in propionate, its final accumulation (2.99 mmol/L) was significantly lower (*p* < 0.05) than that of inulin (4.68 mmol/L). The most striking finding came from the CP group, whose propionate yield (7.32 mmol/L) was significantly higher than that of the inulin-positive control group, highlighting its unique properties that promote propionic acid production. As a consequence, this elevation can be attributed to the molecular structure of CP that is abundant in glycosidic bonds (e.g., β-1,4 or β-1,3 linkages), favoring propionate generation, coupled with its capacity to selectively promote the proliferation and metabolic activity of propionate-producing bacteria, such as *Phascolarctobacterium* and *Dialister* [48]. For the production of butyric acid (Figure 6d), the inulin group remained relatively stable throughout the process, with an average of about 1.22–1.24 mmol/L, indicating that the production capacity of butyric acid in its fermentation system is limited. The LBP group showed a continuous increase in butyrate production, which can be attributed to its unique rat sugar galactosan-I (RG-I) main chain and the structure with Arabic sugar galactose and Arabic sugar side chain, which promotes mutual nutrition among microorganisms. Primary degraders, such as *Bacteroides*, initially broke down these complex side chains into oligosaccharides, which were then utilized by secondary fermenters such as *Faecalibacterium prausnitzii* and *Eubacterium rectale*, ultimately leading to delayed while sustained butyrate production [49]. In comparison, the PGP group showed a gradual increase, and its final butyrate level reached 4.16 mmol/L. The PGP group produced more butyrate. This was mainly because it has a highly branched β-glucan core that is complexed with arabinogalactans. This unique structure created a robust matrix. To break it down completely, microbes needed a longer and more step-by-step process.

Regarding the generation of valproate (Figure 6e), it is worth noting that the concentration of the water group gradually increased from 0.82 mmol/L to 1.13 mmol/L, exceeding the concentration level of the inulin group (always below 1 mmol/L) and the LBP group. This phenomenon may be due to changes in the intestinal microbiota, which begin to decompose substances in the body (such as mucinous proteins or shed epithelial cells) to replace the lack of exogenous carbohydrates. Certain valerate-producing bacteria (e.g., *Clostridium* clusters IV and XIVa) could utilize these endogenous materials for slow metabolism, leading to basal-level accumulation [50]. In contrast, the concentration of valproic acid in the CP group and the PGP group was significantly increased, which can be attributed to their unique high sugar content (Table 2). The elevated neutral sugar ratio provided more abundant and sustained fermentation substrates for specific valerate-producing bacteria in the gut, including *Megasphaera elsdenii* and certain *Clostridium* species, which efficiently synthesized valerate via the acetyl-CoA pathway during later stages of neutral sugar (e.g., glucose, galactose) fermentation.

## 4. Conclusions

This study elucidated that plant-derived polysaccharides with different structural characteristics possessed diverse prebiotic potentials. LBP possessed a medium molecular weight and was enriched with monosaccharides, including Gal and Rha, which together contributed to its elevated uronic acid content. This structural profile not only resulted in significantly higher antioxidant activity compared to CP and PGP but also enhanced butyrate production (3.116 mmol/L) and supported sustained intestinal microbial fermentation. CP, abundant in glycosidic bonds (e.g., β-1,4 or β-1,3 linkages), resulted in the highest propionic acid (1.393 mmol/L) generation, corresponding with the lowest pH value (4.39) after fermentation, while PGP, presenting with the highest molecular weight of a highly branched β-glucan core complexed with arabinogalactans, generated the highest butyric acid production (4.159 mmol/L). Finally, the above research confirmed the high prebiotic potential of LBP, CP and PGP, though prior to advancing to human clinical trials, systematic preclinical evaluations, including assessments of cytotoxicity, bioaccessibility, and validation in animal models, are required to comprehensively determine their safety and in vivo efficacy.

## Figures and Tables

**Table 1:** Monosaccharide compositions of different polysaccharides.

Monosaccharide	LBP	CP	PGP
Rha (%)	21.73 ± 0.3 ^a^	0.44 ± 0.43 ^c^	2.05 ± 0.05 ^b^
Ara (%)	2.49 ± 0.34 ^b^	NA	26.38 ± 0.15 ^a^
Gal (%)	33.08 ± 1.24 ^a^	13.04 ± 0.01 ^c^	19.62 ± 0.23 ^b^
Glc (%)	18.91 ± 0.6 ^c^	70.77 ± 0.69 ^a^	51.60 ± 0.12 ^b^
Man (%)	0.60 ± 0.12 ^b^	14.02 ± 0.87 ^a^	NA
Glca (%)	NA	NA	0.35 ± 0.04 ^a^
Gala (%)	20.78 ± 1.39 ^a^	1.24 ± 1.44 ^b^	NA

Mean values with different letters within the same row were significantly different (*p* < 0.05).

**Table 2:** Chemical compositions of different polysaccharides.

Samples	LBP	CP	PGP
Neutral sugars	46.47 ± 5.03% ^b^	87.05 ± 0.42% ^a^	80.06 ± 0.15% ^a^
Acid sugars	36.76 ± 4.63% ^a^	9.34 ± 0.64% ^b^	6.61 ± 0.12% ^b^
Mw (×10^4^ g/mol)	6.57 ± 0.30 ^b^	3.40 ± 0.15 ^c^	37.92 ± 0.58 ^a^

Mw: weight-average molecular weight. Values were reported as means ± standard deviations (*n* = 6). Mean values with different letters within the same row were significantly different (*p* < 0.05).

## Data Availability

The original contributions presented in this study are included in the article. Further inquiries can be directed to the corresponding authors.
